# Development of an Optimized Two-Step Solid-Phase Extraction Method for Urinary Nucleic Acid Adductomics

**DOI:** 10.3390/biom15040594

**Published:** 2025-04-17

**Authors:** Alexandra Keidel, Jazmine Virzi, Laura Deloso, Carolina Möller, Dale Chaput, Theresa Evans-Nguyen, Yuan-Jhe Chang, Mu-Rong Chao, Chiung-Wen Hu, Marcus S. Cooke

**Affiliations:** 1 Oxidative Stress Group, Department of Molecular Biosciences, University of South Florida, Tampa, FL 33620, USAcarolina@rostonics.com (C.M.); 2 Department of Chemistry, University of South Florida, Tampa, FL 33620, USA; evansnguyen@usf.edu; 3 Department of Molecular Biosciences, University of South Florida, Tampa, FL 33620, USA; chaput@usf.edu; 4 Department of Occupational Safety and Health, Chung Shan Medical University, Taichung 402, Taiwan; 5 Department of Occupational Medicine, Chung Shan Medical University Hospital, Taichung 402, Taiwan; 6 Department of Public Health, Chung Shan Medical University, Taichung 402, Taiwan

**Keywords:** DNA damage, DNA adducts, RNA adducts, adductomics, exposome, DNA repair, urine, mass spectrometry

## Abstract

The exposome represents the totality of endogenous and exogenous exposures across the lifespan. These exposures may result in DNA and RNA damage, in the form of adducts, which is a key factor in the etiology of a variety of human diseases, including cancer. It is understood that, following their repair, nucleic acid adducts are excreted into the urine, making urine an ideal, non-invasive matrix in which to study the whole-body nucleic acid adductome (the totality of nucleic acid adducts). However, the measurement of these adducts in urine presents challenges due to matrix interference and the variety of the chemical nature across the spectrum of nucleic adducts making their “one-size-fits-all” extraction by solid-phase extraction (SPE) challenging. Here, different types of SPE sorbents, and their combination, were evaluated for maximal recovery of nucleic acid adducts from urine. The SPE column combination of ENV+ coupled with PHE provided the best retention of a cocktail of 20 nucleic acid adduct standards. An untargeted high resolution mass spectrometry approach incorporating FeatureHunter 1.3 software was used to demonstrate the ability of this SPE method to successfully recover endogenous urinary nucleic acid adducts in addition to those represented by the cocktail of isotopically labeled standards. Using our approach, FeatureHunter 1.3 recognized approximately 500 adducts in both mouse and human urine samples. Isotopically labeled standards were used to identify a selection of the endogenous adducts and begin the characterization of the urinary nucleic acid adductome of mice and humans.

## 1. Introduction

Damage to nucleic acids (DNA and RNA) can be caused by exposure to a variety of physical, biological, social, and chemical factors [1,2]. These exposures can stem from the external or internal environments, and the totality of all these exposures across the lifespan of an individual is known as the exposome [3,4]. Individual behaviors and lifestyles, such as smoking, diet, or drug use, can lead to the formation of DNA and RNA adducts. For example, the use of tobacco both as cigarettes and as nonsmoking alternatives has been linked to increases in DNA levels of O6-methylguanine, N7-methylguanine, and 8-oxo-7,8-dihydro-2′-deoxyguanosine (8-oxodG), amongst others [5,6]. A diet high in red meat has been linked to cardiovascular disease, where the process of cooking food creates trace amounts of metabolites such as polycyclic aromatic hydrocarbons and heterocyclic amines that, once ingested, cause damage through the alkylation of DNA [7]. Additionally, cooking meat at high temperatures induces the formation of 2-amino-3,8-dimethylimidazo-[4,5-f]quinoxaline, a known human carcinogen which can be found in the body after consumption [8]. Additionally, some chemotherapeutic drugs are DNA damaging agents, in the form of alkylating or crosslinking compounds [9].

DNA adducts may be formed in situ, but they may also be mis-incorporated into DNA as damaged 2′-deoxynucleotide triphosphates (dNTPs) from the dNTP pool. DNA repair helps maintain genomic stability via the removal of these adducts. These repair pathways include nucleotide excision repair (NER) [10] and base excision repair (BER) [11,12,13], as well as the prevention of mis-incorporation of DNA adducts through the activity of dNTP pool sanitizing enzymes such as human mutant homolog 1 (MTH1) [14]. The action of these pathways is understood to result in the excision of the damaged nucleobase (nB) or 2′-deoxynucleoside (2′-dN), which ultimately appears in the urine [10,11].

RNA may also be damaged by many of the same mechanisms as DNA, leading to the formation of homologous adducts [15]. These RNA adducts require repair [16,17], or at least sequestration, to prevent detrimental downstream consequences. Damage to RNA can occur in situ to each of the RNA species, rRNA, tRNA, mRNA, and micro RNA [18]. In addition to direct damage, RNA adducts can be mis-incorporated from the ribonucleotide pool [16]. Since RNA requires single-stranded repair, there is no complementary strand for repair pathways to use, as is the case for DNA [16]. Instead, the YT521-B homology domain-containing protein family (YTHDF) recognizes specific RNA adducts, binds, and marks the strand of RNA for degradation [16,17]. Ultimately these YTHDF protein pathways result in the excision of ribonucleoside (rN) or nucleobase (nB) adducts, which will also appear in urine. Taken together, it is crucial to examine both DNA and RNA to achieve a more comprehensive evaluation of damage to nucleic acids and hence the exposome.

Bio-monitoring these damaging processes and their resulting adducts will help determine genotoxin exposure, carcinogen risk, and personalized medicine. While cellular DNA damage may be directly monitored through tissue analysis, this invasive process requires careful preparation (isolation, hydrolysis) before analysis via high-pressure liquid chromatography (HPLC) coupled with tandem mass spectrometry (*MS*/*MS*) [4]. The results provide information on DNA adducts specific to the cells/tissue from which the DNA was extracted. In contrast, the analysis of urine is a noninvasive means to mitigate these challenges and provides a proxy for the whole-body burden of adducts. HPLC coupled with mass spectrometry (HPLC-MS) is widely used to analyze cellular and urinary DNA adducts and is generally considered the gold standard for such analyses [19,20]. Targeted analysis monitors for specific adducts and, while effective for monitoring known adducts, it cannot reflect the totality of the adductome [21]. Since the adducts repaired from DNA, RNA, and the corresponding (d)NTP pools are present in the urine as nBs, 2′-dN (and rN, in the case of RNA adducts) hydrolysis of the urine is not required before analysis, which is a benefit.

The analysis of urinary nucleic acid adducts does require separation of the analytes from the interfering urine matrix, which includes water, salts, and other molecules that may hinder the analysis of adducts. Failure to remove these compounds will cause matrix effects when analyzing samples by electrospray ionization mass spectrometry, such as a decrease in analyte ion signal due to matrix ion competition [22]. To limit matrix effects, various urinary adduct cleanup approaches are used, such as solid-phase extraction (SPE), for example, using hand-packed polystyrene/polypyrrole columns [23] and hand-packed sulfonic acid poly(glycidyl methacrylate-divinylbenzene)-based microspheres [24]. However, polystyrene/polypyrrole columns are limited to the analysis of 8-oxodG and 8-oxo-7,8-dihydroguanosine [23]. Sulfonic acid poly(glycidyl methacrylate-divinylbenzene)-based microspheres are limited to the analysis of N3-methyladenine, N3-ethyladenine, and N7-ethylguanine [24]. In the present work, we explore combinations of various SPE columns for the isolation of nucleic acid adducts from urine samples. We noted that single-phase SPE was insufficient to isolate the broadest range of nucleic acid adducts; therefore, multiphase SPE was implemented to overcome this problem. By selecting SPE columns with specific sorbents, we leveraged the different chemical interactions between the solid phase and the nucleic acid adducts to retain a wide variety of adducts. To demonstrate a real-world application of the two-phase SPE method, it was paired with our novel FeatureHunter 1.3 software [25] to perform untargeted analysis of representative mouse and human urine.

## 2. Materials and Methods

### 2.1. Chemicals

Stock solution cocktails of 20 standards were generated comprising unmodified and modified nBs, 2′-dNs, and rNs, sourced from a variety of companies (Table 1). Isotopically labeled nBs, 2′-dNs, and rNs were sourced from Toronto Research Chemicals (Table 2). The stock solution was prepared to a final concentration of 10 mg/mL of each standard. Each urine sample was spiked with the stock solution to a final concentration of 1 µg/mL for each standard. HPLC-grade solvents of H_2_O, methanol, and formic acid were obtained from Fisher Chemical (Fair Lawn, NJ, USA), and ammonium acetate was from Sigma Aldrich (St. Louis, MA, USA).

### 2.2. Mouse and Human Urine Sample Collection

Male and female C57BL6/J mice (*n* = 18 of each sex), aged 12–13 weeks, were obtained from Jackson Laboratories (000664) (Bar Harbor, ME, USA) and housed in a certified facility at the University of South Florida compliant with the standards of the Association for Assessment and Accreditation of Laboratory Animal Care International (IACUC approval IS00012003). All mice were randomly assigned and housed in metabolic cages and were acclimated for a minimum of two weeks before use. A maximum of three mice were housed per cage to collect sufficient daily urine samples over a 24 h period. All urine was pooled by sex (15 male and 15 female) to produce a single representative male and female mouse urine sample, which was then aliquoted and stored at −80 °C until use. To confirm that the pooled urine was representative of mouse urine, three pooled male samples and three pooled female samples were omitted from the pooled sample for comparison.

Human urine was collected from volunteers, aged 20 to 30 years, following written informed consent. The study was approved by University of South Florida Human Research Protections (study number 007408). The health of the subjects was determined via a self-reported survey; exclusion criteria included a history of smoking or substance abuse and any history of chronic illnesses. Volunteers were asked to provide at least 5 mL of first-void mid-stream urine [26]. All urine was pooled by sex (nine male and nine female) to produce a single, representative human male and female urine sample, which was then aliquoted and stored at −80 °C until use. To confirm that the pooled urine was representative of human urine, three male samples and three female samples were omitted from the pooled sample and analyzed individually for comparison.

### 2.3. Extraction of Urinary DNA and RNA Adducts by SPE

**Figure 1:**
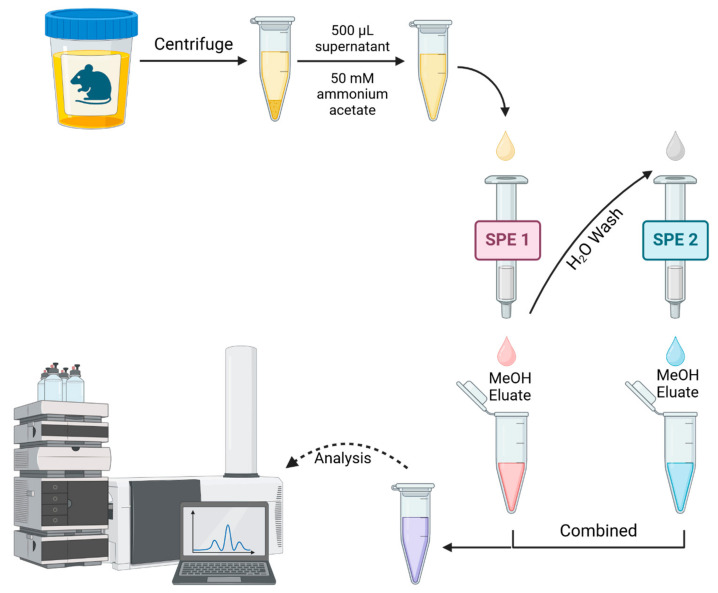
The principle of the multiphase SPE extraction method. The eluent from the initial SPE column was collected and passed through a secondary SPE column with different physicochemical properties from the first. Created in BioRender.com.

To determine the SPE column with the best ability to isolate the DNA adducts, a series of six columns each with different solid phases and chemical properties were evaluated (Table S1). The efficiency of the columns was determined individually before the columns were combined in a sequential SPE method to determine which combination of columns was optimal (Figure 1). Before SPE, aliquots of the urine samples were thawed and brought to room temperature to ensure any precipitated adducts were dissolved [27]. Urine samples were then centrifuged at 16,100× *g* for 15 min to remove any particulates, and the collected supernatant was aliquoted for SPE.

To test the efficiency of each column in isolating DNA adducts from the urine matrix, samples were spiked with a cocktail of 20 standards. A wide variety of adduct standards were chosen to reflect structural diversity amongst adducts found in DNA and RNA. To determine the approximate recovery of each SPE column, one urine aliquot was spiked before SPE while another urine aliquot was spiked after SPE, and both aliquots were then analyzed by HPLC QTOF *MS*/*MS*. By comparing the abundance of the standards in the eluent from the spike before and spike after, the individual recovery of each column and each combination of columns was determined. To ensure that the standard cocktail of adducts did not form secondary products with those present in the urine, the cocktail was spiked immediately before the SPE treatment for the ‘spiked before SPE’ and immediately before analysis for the ‘spiked after SPE’.

Due to the unique nature of the sorbents in each cartridge, each extraction was performed following the manufacturer’s protocol. Broadly, each extraction comprised preconditioning, sample loading, washing, and elution (Figure 1). After the precondition, 500 µL of each urine sample was combined with 500 µL of 50 mM of ammonium acetate, pH 6. For the first extraction (SPE-1), samples were loaded onto the respective cartridges and allowed to filter through. The subsequent washing step was required to remove salt and any interfering matrix that did not bind to the column. However, due to the wide range of adducts and their concomitant chemical properties of adducts, not all of these adducts are recovered by the same sorbent. For this reason, the wash of the first column, which contains the matrix and any missed analytes, underwent a secondary SPE with a different SPE sorbent. In this way, any analytes missed by the first round of SPE should be recovered by the second. The flowthroughs and washes were collected, according to the manufacturer’s protocol, and saved for the second round of SPE. After the elution step, the eluate of the SPE-1 was saved. Both the collected flow-through and the washes from SPE-1 were combined and further extracted using a second type of SPE cartridge with a different sorbent (SPE-2). After following the manufacturer’s protocol for SPE-2, the second eluate was combined with the first eluate. All the samples were evaporated to dryness and reconstituted with 100 µL of 1 mM aqueous ammonium acetate with 0.1% formic acid and centrifuged at 16,100× *g* for 15 min. The supernatants were aliquoted in vials and were then analyzed by HPLC QTOF *MS*/*MS* (Figure 1).

The percentage recovery of each individual column or column combination was calculated by comparing urine samples with standards spiked before SPE to samples with standards spiked after SPE. To ensure there was no artificial inflation of the percent recovery due to endogenous adducts, urine with no added standards was run, and the endogenous signal was subtracted from the spike before SPE and spike after SPE. For this reason, unlabeled standards that may be endogenously present in urine could be used to explore the recovery of the SPE columns without endogenous compounds, increasing the signal of just one sample and thereby skewing the calculated recovery. The cocktail of standards in urine samples spiked before SPE and spiked after SPE were from the same initial standard cocktail, and they were both treated the same beyond the time of the spike.

### 2.4. Time of Flight and Orbitrap Methods for the Analysis of Urinary DNA and RNA Adducts

To identify the optimal combination of SPE cartridges, an optimized HPLC-QTOF-MS method for targeted urinary nucleic acid adductomics was used, based on previously described parameters [20]. The method used an Agilent LC MS Q-TOF 6540 (Agilent Technologies, Santa Clara, CA, USA) with Agilent’s Jet Streaming electrospray ionization (ESI) run in positive mode. The utilized column was a reverse phase Inertsil ODS-3 C18 column (150 × 2.1 mm i.d., 5 μm) from GL Sciences (Tokyo, Japan) with the column temperature set to 40 °C. The aqueous mobile phase (mobile phase A) consisted of 1 mM aqueous ammonium acetate with 0.1% formic acid solution, and 95% methanol (*v*/*v*) containing 0.1% (*v*/*v*) formic acid was the organic mobile phase (mobile phase B). The gradient was established for a run time of 60 min, consisting of 0.5% of mobile phase B at 0–2 min, 30% of mobile phase B at 32 min, and 99.5% of mobile phase B at 48 min with a 3 min hold, and then a 9 min re-equilibration to the initial ratio of the mobile phase. The flow rate was 0.2 mL/min with an injection volume of 20 μL. The analyses were performed using full scan mode, with the mass range restricted from 50 *m/z*–600 *m/z*. For *MS*/*MS*, the acquisition was achieved in auto *MS*/*MS* with fixed collision energies of 10, 20, 40, and 60 V. The software used for data processing and chromatogram peak identification was Agilent (Santa Clara, CA, USA) MassHunter Qualitative Analysis 7.0. Peaks corresponding to each analyte were individually and manually analyzed. The identification of the standards was achieved by considering the retention time, the accurate mass of the precursor and the neutral loss of 116.0473 *amu* corresponding to the loss of the 2-deoxyribose group (2-dR), or 132.0422 *amu* corresponding to the loss of the ribose group in RNA with a mass error tolerance of 5 ppm. Nucleobases were identified from a combination of known retention time, accurate mass, and their individual fragmentation pattern.

To further demonstrate the applicability of our SPE method to untargeted-analysis high-resolution mass spectrometry (HRMS), a Thermo Vanquish Neo UHPLC-Thermo Q-Exactive Plus (Thermo Fisher Scientific, Waltham, Massachusetts, USA) was employed, which is better suited for untargeted analysis. The column we used was a Pep Map RSCL C18 (50 cm × 75 μm i.d., 3 μm) from Thermo Fisher Scientific (Waltham, Massachusetts, USA). The mobile phase consisted of 100% water with 0.1% formic acid solution as mobile phase A, and 100% acetonitrile containing 0.1% formic acid as mobile phase B, with the gradient remaining the same as the TOF method. The flow rate was 0.300 μL/min with an injection volume of 5 μL. For the Q-Exactive plus, the resolution for the full MS was set to 70 K with an automatic gain control (ACG) target of 100 K, and for *MS*/*MS*, it was set to 17.5 K with an ACG target of 20 K. A top 30 data-dependent acquisition was applied with a 30 s dynamic exclusion and a minimum ACG target of 4 K. For *MS*/*MS*, the acquisition was achieved with stepped collision energies of 10, 20, and 30 eV.

### 2.5. Two-Step SPE Validation and Identification of Endogenous Adducts

Replicate mouse urine samples were analyzed using QExactive over the course of three weeks. An ANOVA test was performed to determine any week-to-week variation in the number of analytes recovered. Untargeted analysis with *FeatureHunter 1.3* was used to recover the entirety of the adducts retained by the SPE method. To perform untargeted analysis, the “.raw” data files were converted to “.mzML” files using MS Convert software (Version 3) (Palo Alto, CA, USA). The samples were then analyzed using FeatureHunter 1.3 (Taichung, Taiwan) to extract and tag nucleic acid adducts based on their characteristic fragments (Table 3). Additionally, a cocktail of isotopically labeled standards (Table 2) was used to identify a selection of these endogenous adducts.

### 2.6. Application of Two-Step SPE to the Characterization of Urinary Adductome

To characterize the representative mouse urinary nucleic acid adductome, urine from 15 wild-type mice was pooled by sex, with 15 male and 15 female C57BL6/J mice. This pooled urine was then extracted with the novel two-phase SPE method and run on the Thermo Vanquish Neo UHPLC-Thermo Q-Exactive plus, as described above. An additional sample of urine pooled from three male mice and three female mice was then run for comparison to the pooled urine sample. Pooled and individual samples were extracted and analyzed in triplicate to allow for statistical analysis via volcano plots.

To characterize the representative human urinary nucleic acid adductome, a pooled urine sample from healthy adults aged 20 to 30 years were analyzed in the same way as the pooled mice urine. A total of nine male and nine female volunteers contributed to the pooled urine sample. To establish how representative this pooled urine sample was, three additional samples of male and female urine were used as a determination of variation in an individual’s urine compared to the pooled urinary adductome.

## 3. Results

### 3.1. Extraction of Urinary DNA and RNA Adducts by SPE

**Figure 2:**
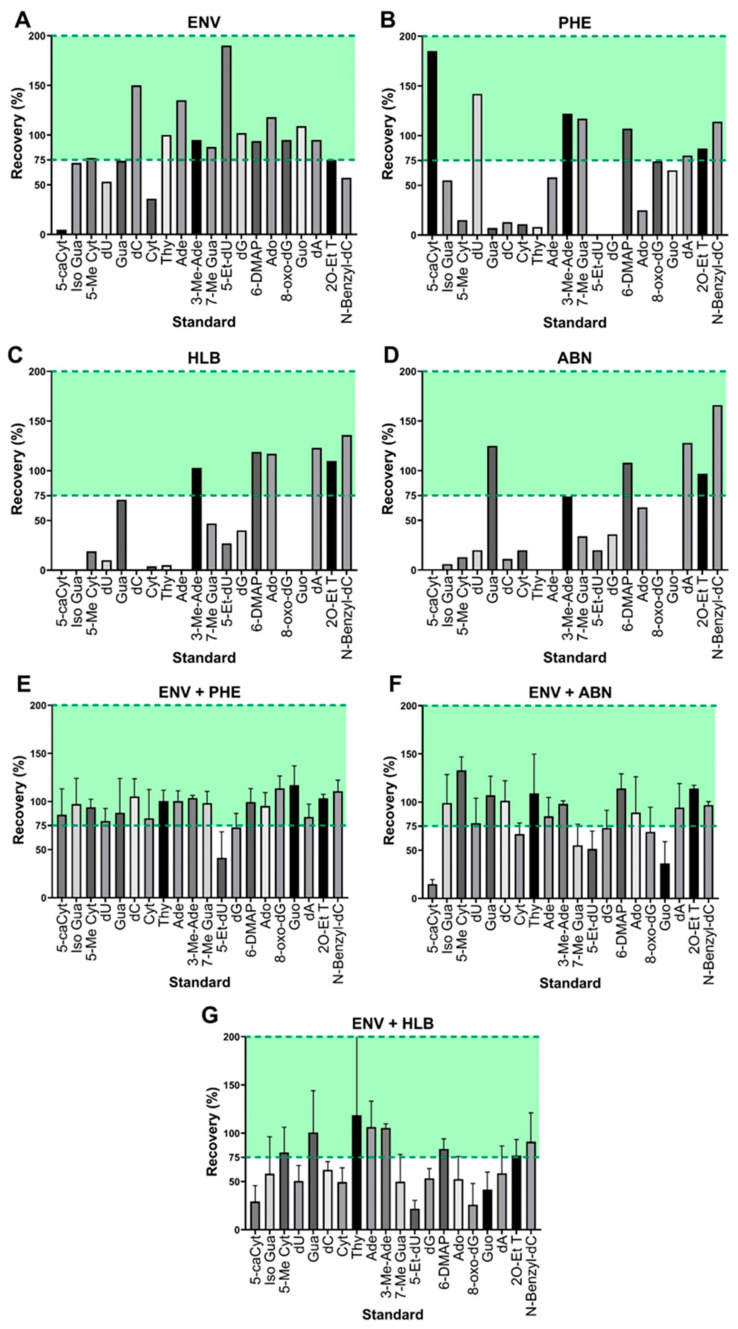
Two-step SPE provides optimal retention across the widest variety of DNA/RNA adduct standards. Standards with a recovery of below 75% were determined to be insufficiently recovered by the SPE column, standards with recovery within 75 to 200% (green) were determined to have been sufficiently recovered by SPE. The SPE columns (**A**) ENV, (**B**) PHE, (**C**) HLB, and (**D**) ABN did not individually have sufficient ability to retain the entire cocktail of spiked standards. While one SPE column was not sufficient for optimal retention, our two-step approach showed higher recovery of DNA/RNA adducts with ENV combined with (**E**) PHE, (**F**) ABN, and (**G**) HLB columns.

We determined that a column would be deemed as having sufficiently recovered the standard if ≥ 75% of the individual standard was recovered. The recovery of individual standards for the SPE columns that separated via reverse phase polar interactions are as follows: the ENV columns recovered 14 out of the 20 spiked standards at over the 75% recovery threshold from the standard cocktail (Figure 2A), whereas the ABN and HLB columns recovered 5 and 6 of the 20 standards, respectively (Figure 2D,C). The CX and AX columns utilized reverse phase cation and anion exchange, respectively. The CX columns recovered 14 of the 20 standards, and AX recovered only 7 of the 20 standards. PHE columns separated via reverse phase aromatic pi–pi interactions and recovered 8 of the 20 standards (Figure 2B). Based on these initial results, the ENV columns were then used as SPE-1 in the two-step combination with the other reverse phase columns. ENV combined with ABN columns recovered 13 of the standards (Figure 2F), and ENV combined with HLB columns recovered 8 of the standards (Figure 2G), whereas ENV combined with PHE columns recovered 18 of the 20 standards (Figure 2E).

**Figure 3:**
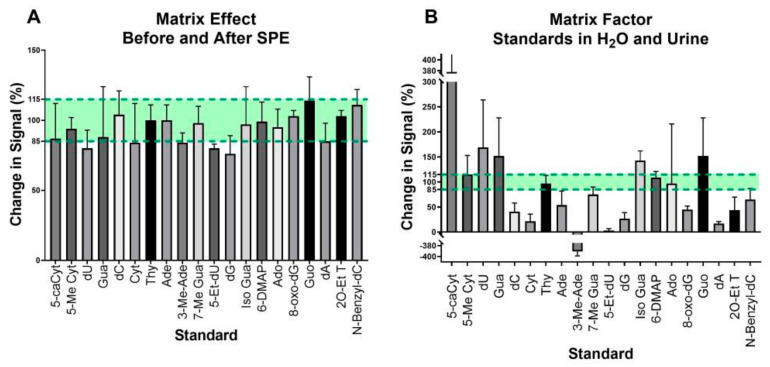
Change in ion suppression for the cocktail of 20 standards due to urine matrix interactions with the matrix for the ENV to PHE SPE method. (**A**) By comparing the ratio of the signal intensity of each standard spiked before SPE to the intensity from spiking after SPE, we were able to determine the ion enhancement or suppression. (**B**) The matrix factor compares the signal of the standards spiked in urine to the signal of the standard cocktail in 1 mM ammonium acetate. Compounds with a change in signal above 100% were considered enhanced while those below 100% were considered suppressed.

The level of ion suppression or enhancement caused by matrix effects was determined through a post-extraction addition method. We compared the ratio of the peaks from the cocktail of 20 standards spiked into a urine sample before SPE with the peaks from the standards spiked post SPE. This approach revealed that 5 of the 20 standards underwent ion enhancement, 3 did not undergo any change, and the remaining 12 standards underwent ion suppression (Figure 3A). Of the five standards that showed ion enhancement, the greatest level enhancement was 114%. Of the 12 standards that showed ion suppression, the greatest level suppression was less than 25%. A comparison of the standard cocktail post SPE and the standard cocktail in 1 mM ammonium acetate gives the matrix factor (Figure 3B). This comparison showed that though we see some improvement in matrix effects in the standard cocktail when comparing across SPE methods, there is still significant matrix interference with the signal when compared to the controlled ammonium acetate sample.

### 3.2. Assay Validation and Identification of Endogenous Adducts

**Figure 4:**
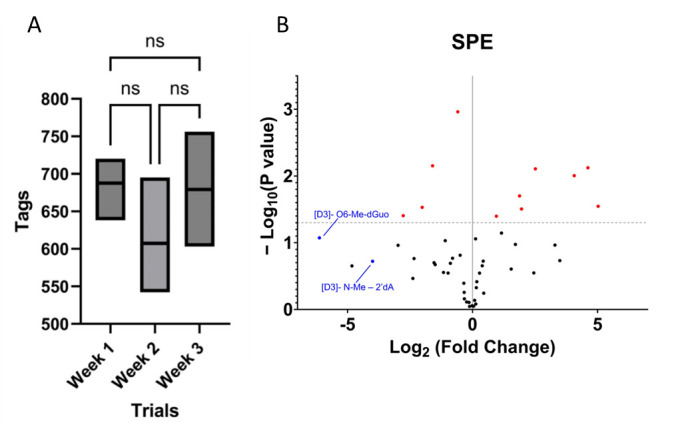
HRMS and SPE are reproducible over the course of three weeks. (**A**) The number of adducts identified in a single sample of human urine run over the course of three weeks showed no significant variation as tested by an ANOVA test to characterize the stability of the instrument method. ns: not significant. (**B**) Initial SPE was replicated three times over the course of weeks to characterize the stability of SPE, and 80% of the adducts (denoted as black dots) did not significantly vary, where as the remaining adducts did significantly varry (denoted as red dotes).

Although *FeatureHunter 1.3* does not directly identify adducts, its ability to assign tags based on characteristic fragmentation patterns allows for the quick identification of adducts that have been previously characterized [25]. Urine samples analyzed in triplicate over the course of three weeks yielded 122 ± 15 of 2′-rN, 157 ± 23 of 2′-dN, and 378 ± 64 of nB adducts that were recovered and tagged by *FeatureHunter 1.3* (Figure 4A). An ANOVA test revealed that the results of HRMS, conducted on the Thermo Vanquish Neo UHPLC-Thermo Q-Exactive plus, did not show significant week-to-week variability in HRMS (*p* value averaging 0.70) in the same urine samples. Additionally, the variation in SPE recovery of the endogenous urinary adducts was characterized by performing SPE on the same 48 urine samples over the course of three weeks. Untargeted analysis of the recovered urinary adducts revealed that 37 out of the 48 urine samples did not significantly vary over the three-week time course (Figure 4B).

**Figure 5:**
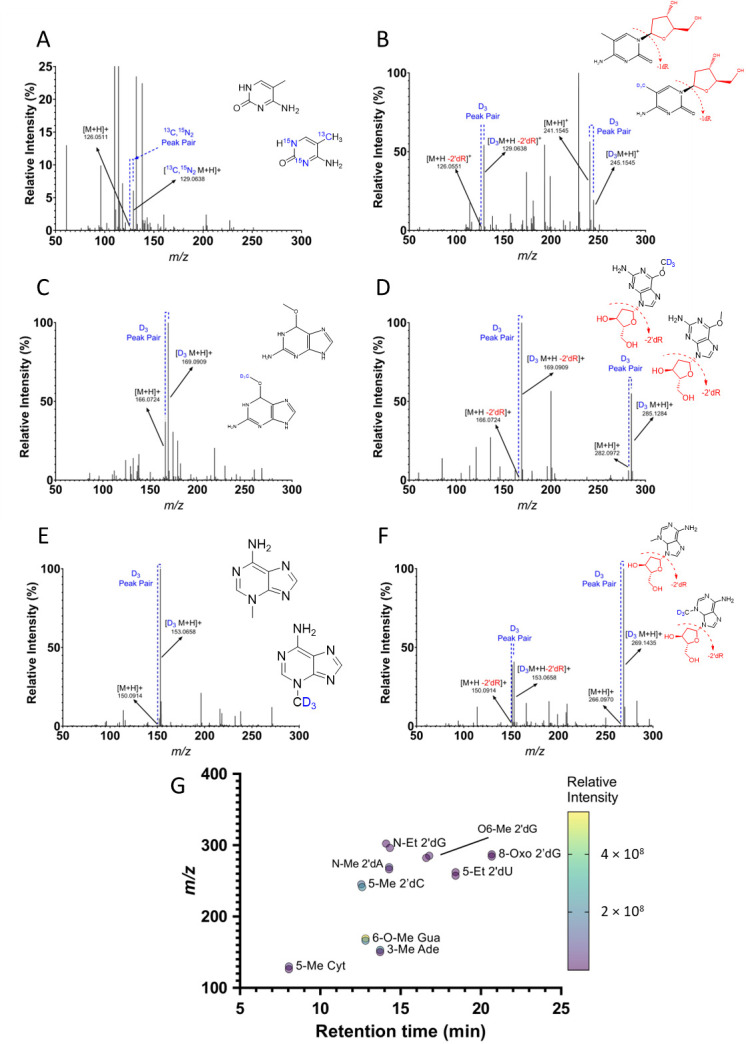
Stable isotopically labeled standards unequivocally identify endogenous adducts in human urine. The labeled standards (**A**) [^13^C, ^15^N_2_]-5-Me-Cyt, (**B**) [D_3_]-5-Me-dC, (**C**) [D_3_]-O6-Me-Gua, (**D**) [D_3_]-O6-Me-dG, (**E**) [D_3_]-3-Me–Ade, and (**F**) [D_3_]-N6-Me-dA were used to identify the unlabeled counterparts present in urine. The relative intensity of the isotopically labeled standard to the unlabeled counterpart is due to difference in concentrations of the standard to that of the endogenous adduct. (**G**) The standards and counterparts were identified through targeted analysis of peak pairs with the characteristic mass difference of the labeled standard and the unlabeled endogenous counterpart. The mass difference for the standards that have an isotopic label of D_3_ was 3.0188 amu, and for those labeled with ^13^C and ^15^N_2_, the mass difference was 2.9974 amu.

Isotopically labeled standards were used to identify their unlabeled counterparts that are present in urine. The identities of the nB and 2′-dN counterparts were determined through the monitoring of the characteristic loss of the sugar giving a set of peaks with a mass difference of 116.0473 *m*/*z* (Figure 5A–F). A targeted search in mouse urine was able to identify all nine labeled standards’ unlabeled counterparts present in urine, as confirmed by their labeled peak pair (Figure 5G).

### 3.3. Characterization of Urinary Adductome

**Figure 6:**
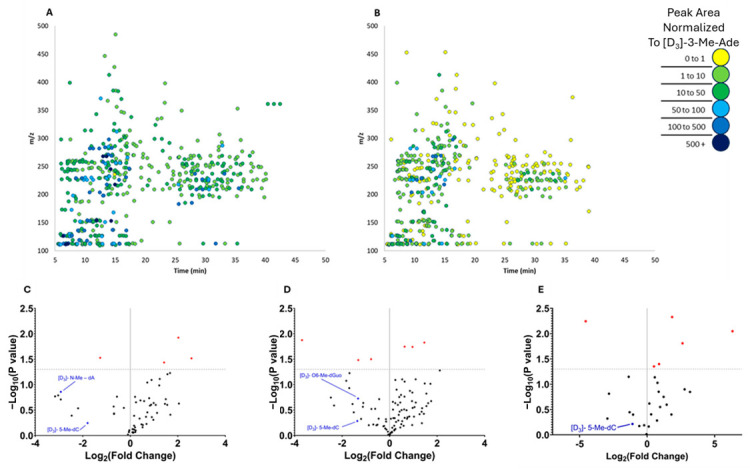
Characterization of the representative urinary adductome of male and female mice. (**A**) A total of 553 adducts were detected by *FeatureHunter 1.3* in the pooled urine sample of 15 female mice. (**B**) A total of 493 adducts were detected by *FeatureHunter 1.3* in the pooled urine sample of 15 male mice. The signal from each adduct was normalized to the internal standard [D_3_]-3-Me–Ade. (**C**) In urine of the individual female mice, of the 62 adducts consistently detected across all samples, 58 adducts did not show significant variation compared to the pooled sample. (**D**) In urine of the individual male mice, of the 99 adducts consistently detected across all samples, 93 adducts did not show significant variation in signal intensity compared to the pooled sample. (**E**) In comparing both male and female mice together, of the 28 adducts consistently detected in both the pooled sample of males and the pooled sample of females, 22 adducts did not show significant variation in signal intensity. The adducts under the horizontal dotted line (denoted as black dots) showed no significant variation between the pooled and individual samples.

Our two-step SPE/HRMS approach was then applied to both human and mouse representative urines to illustrate the urinary DNA/RNA adductome in these species. These characterizations revealed that 553 adducts were present in the pooled representative sample of female mice urine, while male mice urine contained 493 adducts (Figure 6A,B). In female mice, 62 adducts were identified via *FeatureHunter 1.3* across both the pool of 15 female mice and the small pool of three mice. Of these 62 adducts, 58 did not show significant variation between the two pools (Figure 6C). In male mice, 99 adducts were tagged via *FeatureHunter 1.3* across both the pool of 15 male mice and the small pool of 3 mice. Of these 99 adducts, 93 did not show significant variation between the two pools (Figure 6D). When comparing across sexes, in female and male mice, there were 28 adducts discovered in common, and of these, 6 significantly varied (Figure 6E).

**Figure 7:**
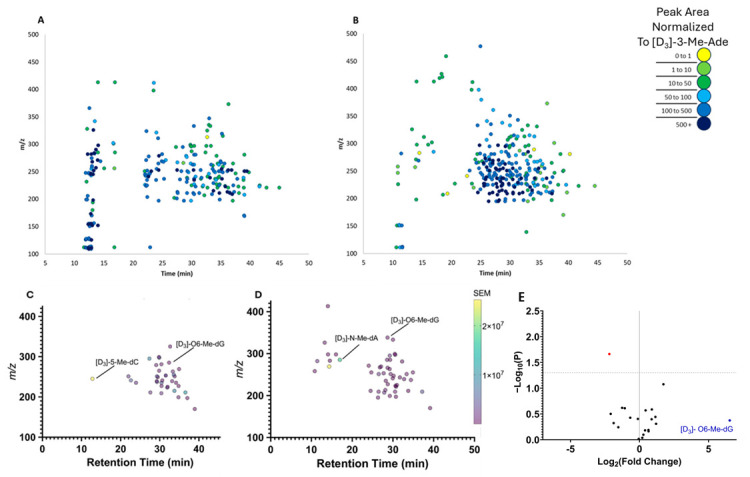
Characterization of the representative urinary adductome of male and female humans. (**A**) In females, a total of 239 adducts were detected by FeatureHunter 1.3 in the pooled sample. (**B**) In males, a total of 270 adducts were detected by *FeatureHunter 1.3* in the pooled sample. The signal of each adduct was normalized to the internal standard [D_3_]-3-Me–Ade. (**C**) In females, there were 37 adducts consistently detected in both the pooled sample and the individual samples. (**D**) In males, there were 49 adducts consistently detected in both the pooled and the individual samples. (**E**) In comparing males and females together, of the 21 adducts consistently detected in both the pooled sample of males and the pooled sample of females, 20 adducts did not show significant variation in signal intensity. The adducts under the horizontal dotted line (black) showed no significant variation between the pooled and individual samples.

In humans, these characterizations (Table 3) revealed that 239 adducts were present in the pooled representative urine sample of nine females, and 270 adducts were present in male humans’ urine (Figure 7A,B). In females, 37 adducts were identified via *FeatureHunter 1.3* across both the pool of nine females and the individual urines of three humans (Figure 7C). In males, 49 adducts were identified via *FeatureHunter 1.3* across both the pooled urine from nine males and the individual urines of three males (Figure 7D). When comparing between sex, there were 21 adducts discovered in common, and of these, 20 did not significantly vary between sex (Figure 7E).

## 4. Discussion

Initially, traditional single-phase SPE was performed with each column to determine the individual column’s ability to retain the analytes of interest. The extraction efficiency of the SPE columns was determined by the recovery of the standards, using the peak intensity/abundance detected by MS, with *MS*/*MS* to confirm identity. The percentage recovery was calculated by comparing a urine sample that had the standards spiked before SPE to a sample that had the standards spiked after SPE. No single column was adequate to recover the complete range of standards. Consequently, the two-phase SPE method developed in this study demonstrated that this approach was effective in enriching the adducts present in urine. Due to the wide variety and therefore chemical properties of adducts, the interactions between the stationary phase and the adducts present in urine in a single SPE column were insufficient to recover the entirety of the adductome. By leveraging a combination of SPE columns with different stationary phases, the adducts that were not retained by the first SPE column may be retained by the second.

The ENV column was selected for the first separation step due to its demonstrated superior recovery capacity. For this reason, the ENV column was used in tandem with other SPE columns to enhance the recovery of the standard cocktail. Based on the individual recovery performance of the columns, the combination of the ENV and CX columns showed promise for achieving a comprehensive recovery. However, adjusting the pH of the solvents to elute the adducts from the column may pose a risk of destabilizing and degrading nucleic acid adducts [28,29]. Therefore, both CX and AX columns were excluded from the selection process thereafter. The remaining columns used ammonium acetate with a pH of 6 to mimic the pH of urine, in line with previous studies [23]. The columns PHE, ABN, and HLB were tested in tandem with the ENV column, and the combination of the ENV followed by PHE columns demonstrated the greatest recovery of adducts. To assess the reproducibility of this two-step SPE approach, replicate analyses of the same urine sample were conducted in triplicate over a period of three weeks, yielding a total of nine analyses.

One limitation of SPE is that the concentration of larger sample volumes may result in higher levels of signal-modifying matrix compounds [30]. To determine whether the matrix effects could be mitigated by SPE, a post-extraction addition method was implemented [30] using our cocktail of standards. We discovered that the matrix effect on ion signals was within acceptable limits, albeit with some enhancement and some suppression. Although the standard cocktail is a limited representation of all the adducts found in urine, it was reassuring to note that levels of ion enhancement did not exceed 125%, and ion suppression did not exceed 25%. However, when comparing the standard cocktail in urine to the cocktail in ammonium acetate, a level of matrix interference remained. To an extent, this matrix interference was expected, as the intention was to have unknown nucleic acid adducts remaining in urine. We subsequently discovered that there were nearly 500 adducts present in the urine samples in addition to those present in the cocktail. It is conceivable that one or more of these nucleic acid adducts in the urine sample may be contributing to matrix interference.

Stable isotopically labeled standards are used to begin to identify the adducts that may be present in a sample based on the mass difference between the labeled standard and its unlabeled counterpart [31]. Considering the retention times of the labeled and unlabeled peak pairs allowed us to identify whether the adduct was in the 2′-dN or nB form. Without the use of labeled standards, overlapping fragmentation patterns would make identification challenging. These labeled standards were used to identify their unlabeled counterparts in urine, to demonstrate recovery, and to begin to identify adducts that are endogenous in urine.

Our group of 15 C57BL6/J mice served as a healthy reference population for identifying the nucleic acid adducts naturally present in their urine. Given that these mice were not exposed to any known genotoxins, the urinary nucleic acid adductome largely represented endogenously derived adducts arising from the internal exposome of C57BL6/J mice, although we do not expect the adductome to be particularly strain-specific—at least for wild type mice. Variation in the nucleic acid adductomes between male and female mice indicated there were endogenous processes leading to the formation of sex-specific adducts.

Similarly, we used pooled urine from nine healthy individuals to generate a representative human urinary nucleic acid adductome. While our exclusion criteria aimed to remove environmental exposures which are likely to include genotoxins, these were free-living individuals, and the urinary nucleic acid adductome is likely to include contributions from exogenously induced adducts. For example, it has been reported that ambient solar ultraviolet radiation contributes significantly to urinary levels of 8-oxodG and 8-oxo-7,8-dihdroguanosine [32]. By taking into consideration the individual’s urinary adductome separate from the pool, we were able to exclude the adducts that may be caused by variation in the exogenous exposome, which in turn may affect the adductome on an individual basis. The lack of variation between the urinary adductome of male and female humans indicated that of the adducts that are typically present, there are few sex-specific adducts arising from the endogenous exposome.

## 5. Conclusions

We discovered that the number of nucleic acid adducts recovered from urine could be improved by using two SPE columns in tandem, allowing us to leverage different column adsorbent properties to retain the widest range of adducts. The combination of the ENV followed by the PHE cartridges provided the best adduct recoveries, resulting in a highly effective clean-up prior to performing urinary nucleic acid adductomics.

Understanding how the exposome contributes to the formation and diversity of nucleic acid adducts is essential for elucidating the environmental and genetic factors that influence disease risk. Performing untargeted analysis of the urinary nucleic acid adductome represents a valuable means to comprehensively and non-invasively assess the body burden of adducts in mice, humans, and any other species from which urine can be obtained.

## Figures and Tables

**Table 1:** Composition of the cocktail of standards used in SPE analysis. A cocktail of the following modified and unmodified DNA/RNA standards was used to determine the adduct recovery for each SPE column. To ensure consistency, a stock cocktail of each standard at 1 mg/mL per standard was spiked into the urine samples to a final concentration of 1 µg/mL.

Standard	RT (min)	*m/z* [M + H]^+^	Abbreviation	Supplier
5-carboxycytosine	2.11	156.0411	5-caCyt	Toronto Research Chemicals (North York, Toronto, ON, Canada)
Isoguanine	5.07	152.0565	Iso Gua	Toronto Research Chemicals (North York, Toronto, ON, Canada)
5-Methylcytosine	5.86	126.0661	5-Me Cyt	Toronto Research Chemicals (North York, Toronto, ON, Canada)
2′-deoxyuridine	5.93	251.0626	dU	Toronto Research Chemicals (North York, Toronto, ON, Canada)
Guanine	6.26	152.0559	Gua	ACROS Organics (Waltham, MA, USA)
2′-deoxycytidine	7.80	228.0974	dC	Toronto Research Chemicals (North York, Toronto, ON, Canada)
Cytosine	7.81	112.0503	Cyt	Thermo Scientific (Waltham, MA, USA)
Thymine	9.03	127.0502	Thy	ACROS Organics (Waltham, MA, USA)
Adenine	10.77	136.0618	Ade	ACROS Organics (Waltham, MA, USA)
3-methyl-adenine	11.85	149.0453	3-Me-Ade	ACROS Organics (Waltham, MA, USA)
7-methylguanine	11.90	166.0726	7-Me Gua	Toronto Research Chemicals (North York, Toronto, ON, Canada)
5-ethyl-2′-deoxyuridine	12.13	275.0623	5-Et-dU	Toronto Research Chemicals (North York, Toronto, ON, Canada)
2′-deoxyguanosine	13.55	268.104	dG	Toronto Research Chemicals (North York, Toronto, ON, Canada)
6-Methylaminopurine	16.97	150.0772	6-DMAP	Toronto Research Chemicals (North York, Toronto, ON, Canada)
Adenosine	17.76	268.104	Ado	ACROS Organics (Waltham, MA, USA)
Guanosine	18.20	284.0997	Guo	ACROS ORGANICS (Waltham, MA, USA)
8-oxo-7,8-dihydro-2′-deoxyguanosine	18.20	284.0997	8-oxodG	SIGMA (Darmstadt, Germany)
2′-deoxyadenosine	18.88	252.1088	dA	Toronto Research Chemicals (North York, Toronto, ON, Canada)
2-O-ethylthymidine	25.06	271.1282	2O-Et T	Chem Cruz (Huissen, The Netherlands)
N-Benzyl-2′-deoxycytidine	40.69	332.2415	N-Benzyl-dC	Toronto Research Chemicals (North York, Toronto, ON, Canada)

**Table 2:** Composition of the cocktail of isotopically labeled internal standards. This cocktail was used with the Orbitrap mass spectrometer to identify unlabeled adducts present in urine. All standards were sourced from Toronto Research Chemicals (North York, Toronto, ON, Canada).

Name	Abbreviation	*m*/*z* [M + H]^+^	Supplier
[^13^C, ^15^N_2_]-8-Oxo-7,8-dihydro-2′-deoxyguanosine	[^13^C, ^15^N_2_]-8-Oxo-dG	287.22	Toronto Research Chemicals
[D_3_]-5-Methyl-2′-deoxy Cytidine	[D_3_]-5-Me-dC	245.26	Toronto Research Chemicals
[^13^C, ^15^N_2_]-5-Methyl Cytosine	[^13^C, ^15^N_2_]-5-Me-Cyt	129.09	Toronto Research Chemicals
[D_3_]3-Methyl Adenine	[D_3_]-3-Me–Ade	153.17	Toronto Research Chemicals
[D_5_]-5-Ethyl-2′-deoxyuridine	[D_5_]-5-Et-dU	258.29	Toronto Research Chemicals
[D_6_]-2′-deoxy-N-ethylguanosine	[D_6_]-N-Et-dG	302.33	Toronto Research Chemicals
[D_3_]-2′-Deoxy-N-methyladenosine	[D_3_]-N-Me-dA	269.29	Toronto Research Chemicals
[D_3_]-6O-Methyl-2′-deoxyguanosine	[D_3_]-O6-Me-dG	285.13	Toronto Research Chemicals
[D_3_]-6O-Methyl-guanine	[D_3_]-O6-Me-Gua	169.08	Toronto Research Chemicals

**Table 3:** Individual tags were used in combination to identify signals that are associated with (A) 2′-dN, (B) rN, and (C) nBs. Individual tags are associated with neutral losses (NL) of common fragments from adducts. Leveraging the tags allows for classification of the adduct in terms of the associated nucleobase and the form of the adducts, whether it is a 2′-dN, rN, or nB.

**(A) Modified 2′-dN**	**-rN**	**[1]–[4⋃7⋃8⋃46⋃47⋃48⋃49]**
Tag	Feature parameter (*m*/*z*)	Feature description
1	116.047344	NL of dR
4	232.094688	NL of 2 dR
7	248.089602	NL of dR + R
8	262.105252	NL of dR + MeR
46	237.067095	NL of Cys + dR
47	262.152872	NL of Lys + dR
48	271.116821	NL of His + dR
49	320.137222	NL of Trp + dR
**(B) Modified rN**	**-R**	**[2]–[5⋃7⋃9⋃50⋃51⋃52⋃53]**
	**-MeR**	**[3]–[6⋃8⋃9⋃54⋃55⋃56⋃57]**
2	132.042258	NL of R
3	146.057908	NL of MeR
5	264.084516	NL of 2R
6	292.115816	NL of 2 MeR
7	248.089602	NL of dR + R
8	262.105252	NL of dR + MeR
9	278.100166	NL of R + MeR
50	253.062009	NL of Cys + R
51	278.147786	NL of Lys + R
52	287.111735	NL of His + R
53	336.132136	NL of Trp + R
54	267.077659	NL of Cys + MeR
55	292.163436	NL of Lys + MeR
56	301.127385	NL of His + MeR
57	350.147786	NL of Trp + MeR
**(C) Modified nBs**	**Gua**	**[78⋃79⋃80]–[115⋃116]b**
		**[22]–[27⋃28⋃29⋃30⋃37]c**
	**Ade**	**[81⋃82⋃83]–[115⋃116]b**
		**[23]–[28⋃31⋃32⋃33⋃38]c**
	**Cyt**	**[84⋃85⋃86]−[115⋃116]b**
		**[24]–[29⋃32⋃34⋃35⋃39]c**
	**Thy**	**[87⋃88⋃89]–[115⋃116]b**
		**[25]–[30⋃33⋃35⋃36⋃40]c**
	**Ura**	**[90⋃91⋃92]–[115⋃116]b**
		**[26]–[37⋃38⋃39⋃40⋃41]c**
22	151.04941	NL of Gua
23	135.054495	NL of Ade
24	111.043262	NL of Cyt
25	126.042928	NL of Thy
26	112.027277	NL of Ura
27	302.09882	NL of 2 Gua
28	286.103905	NL of Gua + Ade
29	262.092672	NL of Gua + Cyt
30	277.092338	NL of Gua + Thy
31	270.10899	NL of 2 Ade
32	246.097757	NL of Ade + Cyt
33	261.097423	NL of Ade + Thy
34	222.086524	NL of 2 Cyt
35	237.08619	NL of Cyt + Thy
36	252.085856	NL of 2 Thy
37	263.076687	NL of Ura + Gua
38	247.081772	NL of Ura + Ade
39	223.070539	NL of Ura + Cyt
40	238.070205	NL of Ura + Thy
41	224.054555	NL of 2 Ura
78	151.049409	PI *m/z* of [Gua]^+•^
79	152.056686	PI *m/z* of [Gua + H]^+^
80	135.030137	PI *m/z* of [Gua + H − NH_3_]^+^
81	135.054495	PI *m/z* of [Ade]^+•^
82	136.061771	PI *m/z* of [Ade + H]^+^
83	119.035222	PI *m/z* of [Ade + H − NH_3_]^+^
84	111.043261	PI *m/z* of [Cyt]^+•^
85	112.050538	PI *m/z* of [Cyt + H]^+^
86	95.023989	PI *m/z* of [Cyt + H − NH_3_]^+^
87	126.042927	PI *m/z* of [Thy]^+•^

## Data Availability

The original contributions presented in this study are included in the article/Supplementary Materials. Further inquiries can be directed to the corresponding authors.

## Supplementary Materials

The following supporting information can be downloaded at: https://www.mdpi.com/article/10.3390/biom15040594/s1, Table S1: SPE columns were used to determine their efficiency in isolating DNA and RNA adducts from urine samples.

## Abbreviations

The following abbreviations are used in this manuscript:

8-oxo-dG	8-oxo-7,8-dihydro-2′-deoxyguanosine
dNTPs	2′-deoxynucleotide triphosphate
NER	Nucleotide excision repair
BER	Base excision repair
MTH1	Mutant homolog 1
nB	Nucleobase
2′-dN	2′-deoxyribonucleoside
YTHDF	YT521-B homology domain-containing protein family
rN!	Ribonucleoside
nBs	Nucleobases
HPLC	High-pressure liquid chromatography
MS/MS	Tandem mass spectrometry
HPLC-MS	Liquid chromatography coupled with mass spectrometry
SPE	Solid-phase extraction
QTOF	Quadrupole Time-of-Flight
HRMS	High-resolution mass spectrometry
