# Supplementary material for: Development of an Optimized Two-Step Solid-Phase Extraction Method for Urinary Nucleic Acid Adductomics
Source: Biomolecules. 2025 Apr 17;15(4):594. doi: 10.3390/biom15040594 (PMC12024844; doi:10.3390/biom15040594)
Supplement: Supplementary file 1 [file biomolecules-15-00594-s001.zip › biomolecules-3471066-supplementary.pdf]

Supplementary Materials

Table S1. SPE columns were used to determine their efficiency in isolating DNA and RNA adducts from urine samples. Each column possesses a unique and different solid phase material, use of two columns ensures a wider range of adducts retained.

Column Name	Manufacturer and Brand	Main retention mechanism
PHE	Thermo—HyperSEP	Pi-Pi interactions
ENV	Biotage—ISOLUTE	Non-polar SPE phase
ABN	Biotage—Evolute Express	Polar (hydrophilic) and non-polar (hydrophobic) interactions
HLB	Waters—Oasis Prime	Extract mid-polar and non-polar compounds
CX	Biotage—Evolute Express	Reverse phase, cation exchange
AX	Biotage—Evolute Express	Reverse phase, anion exchange
